# Facilitators and Barriers of Tai Chi Practice in Community-Dwelling Older Adults: Qualitative Study

**DOI:** 10.2196/42195

**Published:** 2023-01-23

**Authors:** Yan Du, Penny Roberts, Wei Liu

**Affiliations:** 1 School of Nursing University of Texas Health Science Center at San Antonio San Antonio, TX United States; 2 Department of Music Loyola University New Orleans, LA United States; 3 School of Health Professionals University of Texas Health Science Center at San Antonio San Antonio, TX United States

**Keywords:** facilitators, barriers, older adults, practice, adherence, Tai Chi, qualitative study, health outcomes, martial arts, exercise, gerontology, muscle strengthening

## Abstract

**Background:**

Numerous studies have documented the beneficial effects of Tai Chi on a variety of health outcomes, especially in older adults. However, only few studies have examined how to improve the practice and adherence of this Asian-originated exercise among older adults in Western countries.

**Objective:**

This study aimed to identify facilitators and barriers to Tai Chi practice and adherence in community-dwelling older adults.

**Methods:**

This study analyzed the qualitative data collected from 13 participants (mean age 62.0, SD 10.3) at the end of a 15-week randomized controlled trial conducted at a day activity senior center. Semistructured interviews were conducted, recorded, and transcribed; and the data were analyzed using inductive thematic analysis.

**Results:**

Four themes emerged: perceived benefit, threats, facilitators, and barriers. Perceived threats (eg, aging and side effects of medications) and perceived benefits of Tai Chi (eg, balance) inspired participants’ engagement in Tai Chi exercise. On the other hand, barriers to Tai Chi practice and adherence included instructor’s teaching style, the complexity of Tai Chi postures and movements, and existing health conditions (eg, hip problems). In essence, factors like Tai Chi class availability, family and peer support, as well as practicing Tai Chi with music may facilitate Tai Chi exercise adherence.

**Conclusions:**

The study findings could provide valuable information to health professionals, such as nurses and physical therapists, in developing and implementing effective Tai Chi programs in care plans. Considering health conditions, tailoring Tai Chi exercise instruction styles, encouraging social and peer support, and incorporating music may promote Tai Chi practice and adherence.

## Introduction

### Background

The population worldwide is rapidly aging, and the global percentage of adults aged 65 years and older is projected to double by the year 2050 [1]. Aging is frequently accompanied with increased chronic health conditions, including but not limited to osteoporosis, sarcopenia, cancer, heart disease, stroke, diabetes, and Alzheimer disease [2-4]. It is widely evidenced that chronic conditions significantly increase the risk of falls and physical disability, resulting in poor quality of life and premature death [5-8].

Tai Chi, a body-mind practice originating in China, has generated increasing attention from health professionals, including nurses, due to evidence that suggests Tai Chi’s ability to enhance health and well-being indices. A growing body of studies have documented the beneficial effects of Tai Chi on a variety of health outcomes, especially in the older population. The number of Tai Chi studies that are indexed in MEDLINE or PubMed increased from 9 before 1990 to 105 between 1990 and 2003, then rising to 234 between 2004 and 2008, and even higher between 2009 and 2013 to 362 [9]; this number increased to 2336 between 2014 and 2021. The health benefits of Tai Chi practice include but are not limited to physical function [10-13], cardiovascular diseases [14], mental health [15,16], the musculoskeletal system [17,18], balance and fall prevention [13,19], and cognitive function [20,21]. One of the implications of practicing Tai Chi consistently is relative to improved health benefits. For example, a systematic review reports that the frequency of Tai Chi practice is important for fall prevention in older adults [19]. However, like most types of exercise programs, barriers exist that limits adherence to Tai Chi exercise. Understanding these barriers and facilitators becomes essential for health professionals to develop effective Tai Chi interventions that promotes mind-body exercise for optimal health benefits.

### Objective

In spite of the fact that there have been many studies demonstrating numerous health benefits associated with Tai Chi practice, only a small number of studies have looked at the barriers and facilitators involved in the practice. Gryffin et al [22] suggest that inadequate information and teaching style may serve as an obstacle for Tai Chi practice. However, this study did not address the facilitators of engaging in and adhering to Tai Chi practice. Another study found that encouragement from social supports is a factor that motivates older people to start practicing Tai Chi, and subsequent positive health outcomes from the exercise program can help motivate people to continue practicing Tai Chi [23]. This study was conducted in Taiwan [23]; therefore, the results may not be generalized to individuals living in the Western countries. In addition, even though Tai Chi has been proven to provide health benefit to certain patient populations, we are not aware of any examples of facilitators and barriers in the African American community as it relates to Tai Chi. The objective of this study was to explore the facilitators and barriers of Tai Chi practice and adherence in both White and African American older adults.

## Methods

### Study Participants

Our study reports the findings of the qualitative data collected at the end of a 15-week randomized controlled trial, which assessed the effects of practicing Tai Chi with music on fall-related factors. The trial was conducted in the fall of 2014 at a day center in the Southern United States, offering a variety of creative arts and activity programs for adults aged 50 and older. A total of 13 women were enrolled, and block randomly assigned into a Tai Chi practice with music group or Tai Chi practice without music group. A detailed study design of the randomized controlled trial was documented in early reports [24].

### Ethics Approval

This study was approved by the Tulane University Institute Review Board (#630231). Written consents were obtained from all participants.

### Data Collection

At the end of the 15-week Tai Chi exercise intervention, a semistructured interview was conducted with each of the 13 study participants. An interview guide was developed based on the Health Belief Model, which consists of the following 6 concepts: perceived susceptibility, perceived severity, perceived benefits, perceived barriers, cues to action, and self-efficacy (Multimedia Appendix 1) [25].

Health Belief Model is a theory designed to predict health behaviors to promote good health outcomes [26]. This has been used frequently in nursing to identify factors relative to positive behavior changes [27]. This model suggests that an individual’s perception about health problems, perceived benefits of intervention and barriers to intervention, self-efficacy, and cues to action explains engagement (or lack of engagement) in health-promoting behaviors [25].

The semistructured interview questions were organized in the following segments: (1) perceived susceptibility and severity of health issues occurring with aging, (2) motivations of Tai Chi practice, (3) perceived benefits of Tai Chi practice, and (4) perceived facilitators and barriers for Tai Chi practice. Open ended questions such as “What made you sign up for Tai Chi class?” and “Was there anything that stopped you from practicing Tai Chi?” were included in the interview. Probe questions were further asked when appropriate or deemed necessary to explore participants’ experience and perceptions of Tai Chi practice. Two trained graduate students conducted the semistructured interviews, and each interview lasted about 15-30 minutes. All interviews were audio-recorded and transcribed.

### Data Analysis

Data were analyzed using NVivo (version 8.0; QSR International). Although the Health Belief Model was used to develop the interview guide, the data were analyzed using inductive thematic analysis—a method of identifying, analyzing, and reporting patterns (ie, themes, topics, and ideas) within data without predetermined themes to guide coding processes [28,29]. First, 2 researchers immersed themselves in the qualitative data to become acquainted with the content; throughout, they made notes, comments, and ideas of coding the data. Second, the two researchers independently coded the 13 interviews using open coding, and then the researchers gathered together to reconcile code differences in their respective analyses. Coding discrepancies were discussed between the two researchers until a consensus was reached. Third, one researcher grouped the codes into themes based on the similarities and differences of the codes and cited relevant quotes for each theme, while the other researcher reviewed the themes created, and then both discussed to reach an agreement as different opinions arose.

### Trustworthiness

The trustworthiness related to credibility, transferability, dependability, and confirmability was enhanced through various approaches starting at the study design stage [30,31]. For instance, prolonged engagement at the study site, member checking, and team meetings were used to improve credibility. Even though the study was conducted at a single senior site, it included both White and African American participants, which might improve the transferability, given that little is known about African Americans regarding this topic. Audio recordings of all conducted interviews were adopted to increase dependability. Confirmability was enhanced through approaches such as coding the data independently with 2 researchers.

## Results

### Sample Characteristics

Table 1 shows characteristics of the study participants. Participants in the study were female, aged 50 to 84 years, with an average age of 69.2 (SD 8.5) years. Half of the participants were African American. This was a group of relatively high educated adults, with 82% (11/13) having higher than high school education. Only 23% (3/13) still worked full- or part-time. Around 46% (6/13) of the participants either were married or lived with a partner. The average reported exercise hours per week was 4.5 (SD 2.1) hours, including walking, yoga, ballet, and strength training (not shown in Table 1). In total, 61% (9/13) of the participants had practiced Tai Chi before this study, mostly for one semester and at the same facility as in this study, with a different volunteer instructor. The average class attendance rate for the clinical trial was 71%.

**Figure 1:**
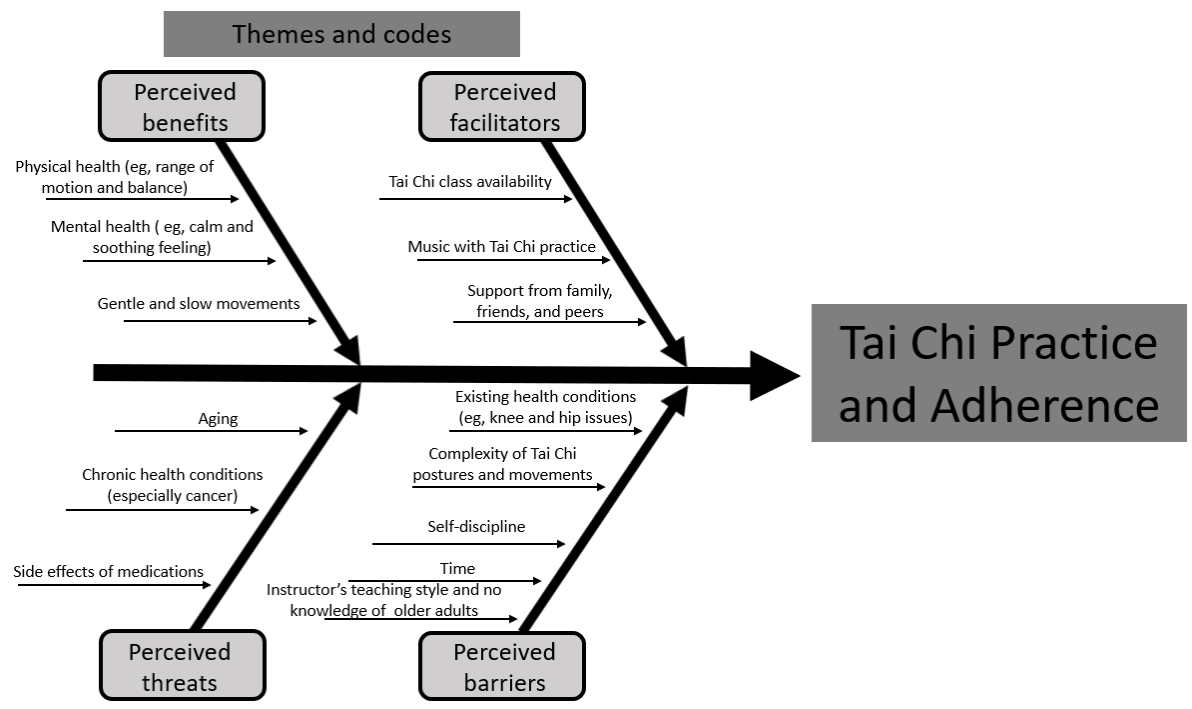
Themes and codes for Tai Chi practice and adherence.

Four major themes related to this study topic emerged from the qualitative interviews, as follows: perceived threats, perceived benefits, perceived facilitators, and perceived barriers for Tai Chi practice and adherence. The corresponding codes for each theme are displayed in Figure 1.

**Table 1:** Characteristics of participants (N=13).

Variables		Values
Age (years), mean (SD)		69.2 (8.5)
**Race, n (%)**		
	White	6 (46)
	African American	7 (54)
**Education, n (%)**		
	High school (yes)	2 (18)
	>High school (yes)	11 (82)
**Work status, n (%)**		
	Not working	10 (77)
	Part- or full-time	3 (23)
**Marital status^a^, n (%)**		
	Married	6 (46)
	Other	7 (54)
Previous Tai Chi (yes), n (%)		9 (61)
Exercise hours/week, mean (SD)		4.5 (2.1)
Compliance rate, mean (SD)		0.71 (0.23)

^a^Marital status: married=married with spouse alive; other=widow, single, or divorced.

### Perceived Threats

The perceived threats due to aging were typical reasons that inspired participants to engage in exercise, including Tai Chi. One of the oldest participants in the study stated the following:

You have to keep at it [exercise- including Tai Chi]. That’s how I feel. At my age, if I don’t, you lost a lot.

When talking about what drives Tai Chi practice, some participants responded with a combination of reasons related to aging and chronic health conditions, like one participant said:

I suffer with blood pressure and I’m getting up in age.

In particular, cancer was frequently repeated as a health threat in this study population. One cancer survivor said the following:

One of the reasons why I’m so conscientious about trying to do different forms of exercise and keep myself really healthy is because I do not want a recurrence.

Relatedly, the side effect of drugs was also motivation for these reasons. For example, another cancer survivor responded:

Yeah so you know, really, the cancer is, that’s the primary, that’s my primary health challenge. Everything else is pretty good shape but because of the drugs that I take there are these rippling effects, it’s one of the reasons why I try to stay active.

### Perceived Benefits

Perceived health benefits consist mainly of two aspects: the benefits they learned from scientific report or other sources; and the benefits they experienced themselves. Perceived benefits from other sources were usually stated as the motivation to start Tai Chi practice. Participants described appreciation of Tai Chi in a variety of ways. Most participants stated that they had prior first- or second-hand knowledge of Tai Chi. Many were aware of the reported health benefits of Tai Chi, particularly balance improvement. For example, one participant in response to being asked about reasons for wanting to take part in Tai Chi, said the following:

Undoubtedly the benefits. As I said in class yesterday, range of motion issues, balance issues, um, focus, concentration.

Additionally, as they started to practice Tai Chi exercises, experienced benefits were stated as factors that facilitate the continuity to practice and adhere to Tai Chi. In addition to frequently perceived balance improvement, participants also mentioned the psychological aspect they gained from the movements, such as the following statement by a participant:

Um, I don’t only like the movements; I also like the soothing aspect.

Likewise, characteristics of Tai Chi movements were perceived positively by participants. The gracefulness of the movements was especially attractive; In that regard, a participant said the following:

I have seen Tai Chi through the years, and it just looked so beautiful; the movements looked so beautiful that to get some degree of grace would be wonderful for me.

The slowness of the movements was also acknowledged; one participant, after stating that she had some health issues and considered her age, mentioned,

I wanted to do something that wasn’t like being out running or something strenuous, so I decide to

do Tai Chi

### Perceived Barriers

Similar to participating in other exercises, self-discipline and time management are among the most common barriers for engaging in Tai Chi practice, with several participants mentioning self-discipline as the most challenging barrier that kept them from adhering to Tai Chi class schedules. For instance, one participant said the following:

I think, personally, I just need to discipline myself. It’s more about disciplining myself…That’s why I tried to be as faithful as I can to come to the classes here.

Time was another factor several individuals talked about, as one participant said:

One thing is to make the time to do it. See, I took two other exercise classes, and I am supposed to not have to work anymore, but I do, and so I have to fill in different things.

The complexity of Tai Chi postures and movements were likewise identified as barriers by the participants. Several participants stated it was difficult for them to master movements. For example, one participant said the following:

For me, right now, that’s what’s throwing me off. I’m not getting the hand movements. She [the instructor] is doing this [movement] and I’m doing that

other movement

Furthermore, finding the right instructor with an appropriate teaching style for the older population was also a barrier. One participant stated the following:

Now I have to say that the first experience I had with taking Tai Chi, it was this really young girl, she must’ve been like 19 or 20 and I think she didn’t realize that we were senior citizens and she needed to not be as intense with it as she was. We kept saying, she forgets that we are not young like she is, we can’t really do all this.

Some subjects perceived multiple barriers at the same time. A participant stated:

Need an instructor in front of me. Just discipline to do it, and sometimes, time was strained.

Lastly, some existing health conditions also restricted participants from practicing, such as hip and knee issues. One participant said the following in this regard:

[B]ecause my hip does give me problems, so you know, with the walk, that’s why I never did Tai Chi. I have to balance on one foot so that hip really is not; the hip and the toe on that one foot doesn’t allow me to balance as good on that left foot.

### Perceived Facilitators

Facilitators included class availability, music with Tai Chi practice, and support from family, friends, and peers. Availability of classes can also promote Tai Chi practice or adherence. One person stated, “I would commit to the class if I had a class (available to me).” In this study, music was added as another component to increase motivation with Tai Chi practice, and participants in the Tai Chi group with music component indicated that music helped them focus and enjoy more of the practice:

I have enjoyed having the music in class. I think it helps me focus. I think that the music has that same ability, so you hear the music and all of the sudden, it’s focused, certainly, but it’s also sets up a criterion for what you’re going to be doing. It brings about kind of an automatic response in the body.

Similarly, all participants enrolled in the Tai Chi without music group wanted to add music in the future when practicing Tai Chi.

In addition, peers, friends, and family members were among the most common facilitators for adhering to Tai Chi exercise, with one participant commenting the following:

And because I feel like I’m getting older my brain is, you know, really getting jelly-like. So, it took me a while to catch up on to Tai Chi.…And I would tell XX (a participant in Tai Chi class), ‘XX, I think I’m going to drop this class, and she would say, ‘No, don’t drop it, you’re not doing any worse than the rest of us!’

One member of the Tai Chi classes reported:

My daughter wants me to do this, because it’s good for me.

## Discussion

### Perceived Facilitators and Barriers

This qualitative study identified several facilitators (eg, practicing with music and class availability) and barriers (eg, lack of quality instructors and complexity of Tai Chi movements) of Tai Chi practice and adherence perceived by community-dwelling older adults. These findings are important for nurses and other health care professionals to develop and recommend effective Tai Chi programs and interventions for older adults’optimal health benefits.

Perceived health conditions and aging were the two major motivators for Tai Chi practice, and perceived health benefit of Tai Chi was another motivator for Tai Chi practice. This is consistent with the literature suggesting that perceived threats of health could be a motivator for engaging in healthy lifestyle behaviors [32] and Tai Chi practice [33]. Previous studies have widely documented the health benefits of Tai Chi practice in the older population [11,19,34,35], especially because of its gentle and slow movements [22]. Meanwhile, certain health problems, such as major physical disability, might be an obstacle for engaging in Tai Chi exercise. Fortunately, Tai Chi exercise can be modified and tailored to individuals with physical limitations. For example, the wheelchair Tai Chi includes modified exercises for participants in wheelchairs and has been proven effective for people with disabilities [36]. Therefore, providing tailored instructions and recommendations to target populations is warranted to improve Tai Chi practice and adherence.

In addition to similar barriers to participating in other exercises, such as time restriction and self-discipline [37], participants perceived some unique challenges when practicing this Eastern exercise. First, unlike other exercise, Tai Chi classes are not always available in the community. Even though learning from videos is possible, it is very different from learning on site, especially considering that Tai Chi movements have a substation stretching and turning of the body, which may be difficult to perceive over video and easier to learn with the assistance of an instructor. This could be one of the reasons for retaining participants in Tai Chi practice and Tai Chi studies [38].

Second, Tai Chi was considered both physically and cognitively challenging by the participants. Tai Chi’s origin is from martial arts, and it is an intricate combination of individual head, hand, arm, leg, ankle, upper body, and lower body movements. Tai Chi involves continuous, slow, and rhythmic dynamic loading and unloading with the ability to gradually modify the difficulty of the task, all of which is needed for joint health. Current Tai Chi research can be divided into those analyzing the practice and those that introduce Tai Chi movements, further analyzing their therapeutic effects on particular maladies. Tai Chi, whether performed as an exercise or woven into daily life for fall prevention, is beneficial to the body without causing secondary problems, especially to the joints. Tai Chi has several different styles, including but not limited to the Chen, Yang, Sun, and Wu styles [39]; some of these styles are more physically and cognitively challenging than others. If instructors do not consider older adults’ physical and cognitive changes, Tai Chi exercise may be unnecessarily taxing for this population and deter them from practicing it. In addition, the selection of Tai Chi forms is critical to the success of Tai Chi as a therapeutic intervention; thus, it is crucial that a more precise estimate of joint movement within Tai Chi forms be incorporated into future studies to understand how Tai Chi can optimize joint kinematics and kinetics, then identify the biomechanical mechanisms and their association with different Tai Chi forms. Therefore, instructors who teach Tai Chi to older adults could select the most optimal forms and movements to maximize Tai Chi’s benefits and minimize its harms. It would also be beneficial to standardize the training process for Tai Chi instructors in both future research studies and general practice in the community.

In addition to instructor’s teaching style, music may also play a critical role in influencing Tai Chi learning and adherence. Studies have documented improved learning occurring when music is paired with movements in the music therapy technique of entrainment. Entrainment occurs when music is paired with an activity, further described as “a temporal locking process in which one system’s motion or signal frequency entrains the frequency of another system” [40]. Via this principle, linking movement to rhythm may establish a kinetic pattern that is easier and faster, increases confidence, and therefore, promptly leads to increased compliance in attendance. Although teaching long and complicated movement patterns is traditionally taught with “chunking” (ie, grouping together chunks of information and focusing on one chunk at a time), compound cues may actually improve acquisition [41]; and the addition of music, therefore, encodes basic movements and facilitate progression to difficult patterns.

Lastly, this is one of the first studies to include African American participants in the study of Tai Chi practice. Perceived facilitators and barriers of Tai Chi practice among African American participants were similar to those of their counterparts. Even though the longevity of African Americans is increasing, they generally undergo more chronic conditions and have a higher risk of disability [42,43], which may be improved with Tai Chi. Literature supports the health benefits of Tai Chi practice [9]; therefore, it is important to conduct further studies with larger samples and thoughtful research designs to examine Tai Chi pratice in African American pupulation and other minority groups.

### Limitations

There were a few limitations to this study. One of the limitations was that all study participants were from a single senior center and were previously enrolled in a Tai Chi class. In addition, all our participants reported having at least high school education or a higher level of education. Therefore, the generalizability of the study findings to other populations is limited, and studies that include a diverse population are still needed.

Data from this study were collected in 2014, and despite there being a few other studies examining similar topics since 2014 [22,44], our study population included White and African American participants, which resulted in some unique findings. For example, we found that using music may promote Tai Chi learning experience and Tai Chi practice adherence among this racial diversity, which would be very helpful in implementing Tai Chi in the community settings, particularly; in the face of increasing evidence that reveals the health benefits of Tai Chi exercise in older adults, little is known regarding how to disseminate Tai Chi to diverse older populations. Thus, these results are worthy of being reported and publicized, as this would help guide the development of Tai Chi programs, and it will benefit the aging community.

Nurses play an essential role in health promotion, educating the public and patients on the prevention and management of health conditions, providing evidence-based care and support, advocating for health-related programs and policies, as well as advancing nursing care through research. Tai Chi, as a mind-body exercise, can be practiced in various community settings, including but not limited to hospitals, senior communities, clinics, and nursing homes. The study findings provide valuable information for nurses to develop or identify effective Tai Chi programs to improve health outcomes in older adults. In addition, research exploring strategies to tailor Tai Chi programs to promote Tai Chi practice in populations with different health conditions and background is needed.

### Conclusions

This study found that perceived aging, health issues, and health benefits were common reasons for choosing to practice Tai Chi. Importantly, the barriers to its practice and adherence (eg, lack of quality instructors) need to be addressed; and facilitators, such as practicing with music and class availability, need to be promoted. Although studies have been trending upward about the health benefits of Tai Chi constantly, most of them are very limited in terms of translational forethought. Therefore, research in exploring the dissemination and promotion of Tai Chi exercise is warranted. For instance, strategies must be explored to address the shortage of qualified instructors and train them to meet specific health needs, especially for older adults. Additionally, incorporating music into Tai Chi may reduce anxiety and promote adherence to Tai Chi practices.

## Appendix

Multimedia Appendix 1 Interview guide.
