# Supplementary material for: Facilitators and Barriers of Tai Chi Practice in Community-Dwelling Older Adults: Qualitative Study
Source: Asian Pac Isl Nurs J. 2023 Jan 23;7:e42195. doi: 10.2196/42195 (PMC9976991; doi:10.2196/42195)
Supplement: Multimedia Appendix 1 [file apinj_v7i1e42195_app1.docx]

**Interview Guide**

Hi, I am______, one of the researchers of Tai Chi study. As you already know the purpose of this study from consent form, below is a list of information that we want to know about you. We appreciate your corporation.

a. Perceived susceptibility and severity of health issues along with aging

- Health issues you may have right now or you think you might be susceptible?

______________________________________________________________________

- Do you think these health issues effects are mild, moderate, or serious for you?

______________________________________________________________________

b. Tai Chi practice history and behaviors

- What drives you to practice Tai Chi?

____________________________________________(Like health benefits? interested in practice, etc.)

- How long have you practiced Tai Chi (including the past 15 weeks)?

_______________________________________________________________________

- Where do you usually practice Tai Chi?

_______________________________________________________________________

- - Do you also practice at home by yourself?

_______________________________________________________________________

- - Do you practice alone, in a group, or both?

_______________________________________________________________________

- Do you practice it regularly? How often?

_______________________________________________________________________

c. Do you think your balance has been improved? If so, would you please describe how you think it has been improved or why you think it has been improved?

_______________________________________________________________________

d. Other perceived benefits you may have received from Tai Chi practice? __________________________(Like Physical, mental, social, and other).

e. What are the facilitators and barriers of practicing Tai Chi regularly?

________________________________________________________________________

- Memory
  - Did you have trouble remembering the forms?

_______________________________________________________________________

- Technology
  - Did you have trouble with the CD, DVD, or YouTube video?

_______________________________________________________________________

- - Did it help you to have a visual aid for practicing outside of class?

_______________________________________________________________________

What else may facilitate or create an obstacle to you practicing Tai Chi?

_______________________________________________________________________

f. Practice Tai Chi with music

- If not, imagine what might be the differences of practice Tai Chi with and without music?

_______________________________________________________________________

- If yes
- Do you like to practice Tai Chi with music?

_______________________________________________________________________

- How do you feel when you practice Tai Chi with music? (Exercise time, physical, mental, concentration, etc.)

_______________________________________________________________________

- What are the differences between practicing Tai Chi with and without music?

_______________________________________________________________________

- What kinds of music do you like best to accompany Tai Chi practice?

_______________________________________________________________________

- - Had you ever used music with Tai Chi before this class?

_______________________________________________________________________

- How do you think music improves or hinders your Tai Chi practice?

______________________________________________________________________

- - Does it provide structure, motivation, pace, or something different?

_______________________________________________________________________

- - Do you find it distracting or focusing?

_______________________________________________________________________

- If you continue to practice Tai Chi, will you use music?

_______________________________________________________________________

g. Do you have any other comments or concerns you want to share with us about your experience of practicing Tai Chi?

_________________________________________________________________

Thank you very much for your participation!
